# Length-dependent anisotropic scaling of spindle shape

**DOI:** 10.1242/bio.201410363

**Published:** 2014-11-21

**Authors:** Sarah Young, Sébastien Besson, Julie P. I. Welburn

**Affiliations:** 1 Wellcome Trust Centre for Cell Biology, School of Biological Sciences, University of Edinburgh, Edinburgh EH9 3BF, Scotland, UK; 2 Centre for Gene Regulation and Expression, College of Life Sciences, University of Dundee, Dundee DD1 5EH, Scotland, UK

**Keywords:** K-fiber, Clasp, Microtubules, Mitosis, Spindle

## Abstract

Spindle length varies dramatically across species and during early development to segregate chromosomes optimally. Both intrinsic factors, such as regulatory molecules, and extrinsic factors, such as cytoplasmic volume, determine spindle length scaling. However, the properties that govern spindle shape and whether these features can be modulated remain unknown. Here, we analyzed quantitatively how the molecular players which regulate microtubule dynamics control the kinetics of spindle formation and shape. We find that, in absence of Clasp1 and Clasp2, spindle assembly is biphasic due to unopposed inward pulling forces from the kinetochore-fibers and that kinetochore-fibers also alter spindle geometry. We demonstrate that spindle shape scaling is independent of the nature of the molecules that regulate dynamic microtubule properties, but is dependent on the steady-state metaphase spindle length. The shape of the spindle scales anisotropically with increasing length. Our results suggest that intrinsic mechanisms control the shape of the spindle to ensure the efficient capture and alignment of chromosomes independently of spindle length.

## INTRODUCTION

The self-organization and maintenance of a bipolar spindle is critical to ensure chromosome alignment and the equal distribution of genetic material to the daughter cells during mitosis. The mitotic spindle is composed of dynamic microtubule polymers. These microtubules require a range of associated proteins to allow self-organization into a dynamic diamond-shaped spindle structure. The formation and geometry of the spindle are critical to allow it to correctly segregate the chromosomes. However, amongst eukaryotes there is an impressive cytological diversity in spindle architecture that is governed by intrinsic and extrinsic factors to optimize spindle function (Dumont and Mitchison, 2009b; Goshima and Scholey, 2010). For example, spindle architecture varies dramatically from budding yeast, which have only one microtubule that connects to each chromosome, to *C. elegans*, which have holocentric chromosomes in which centrosomal-nucleated microtubules attach along the length of the chromosomes. Cell volume plays a defining role in spindle shape scaling in *Xenopus*, with increasing concentrations of microtubule regulators also altering spindle size without changing its overall shape (Good et al., 2013; Hazel et al., 2013; Reber et al., 2013). Spindle shape also appears to change during development and across species due to the developmental and differential expression of microtubule-associated proteins (Courtois et al., 2012; Hara and Kimura, 2013; Loughlin et al., 2011). The spindle is diamond-shaped, in both bound regime, where the spindle is constrained by the cell cortex and unbound regime, where the cell volume is much larger than the size of the spindle and does not restrain the spindle, suggesting cell boundaries do not dictate the shape of the spindle. However, how spindle shape is determined remains ill-defined. The scaling of spindle length has been more extensively studied and varies greatly during early development and across species (Pereira and Maiato, 2012; Wühr et al., 2008). There are also differences in spindle length among closely related species and different cell types within species, due in part to the presence, absence, or relative varying levels of microtubule regulators in the cytoplasm (Helmke and Heald, 2014; Loughlin et al., 2011; Wilbur and Heald, 2013). Distortion of spindle geometry has been linked to compromised genome stability and lagging chromosomes in anaphase (Ganem et al., 2007; Storchová et al., 2006). Thus, understanding the rules that govern spindle geometry is key to understanding the function and plasticity of microtubule self-organization in cytologically diverse spindles.

Here we investigate the regulation of spindle shape during spindle formation in a quantitative manner. We report that the shape of the spindle is linearly and anisotropically correlated with spindle length at steady-state. We find that the regulators of microtubule dynamics Clasp1, Clasp2, the chromokinesin Kid, and the kinesin-8 Kif18a contribute to the kinetics of bipolar spindle formation and spindle geometry. Our data indicate that kinetochore-fiber dynamics, as well as polar ejection forces, play a determining role in setting correct spindle shape. Taken together, we propose that spindle shape is fine-tuned by the presence and levels of cytoplasmic microtubule regulators to stabilize the spindle and to ensure that the resulting spindle geometry optimal for high fidelity chromosome capture and segregation.

## RESULTS

### Clasp1 and Clasp2 regulate the steady-state spindle geometry

An extensive cytological diversity in spindle architecture is achieved to support species-specific requirements and to ensure correct chromosome segregation across species. To probe how spindle length and shape is set during spindle elongation, we analyzed the dynamics of spindle geometry formation. We arrested U2OS cells stably expressing mCherry-tubulin in a monopolar configuration after nuclear envelope breakdown by treatment with the Eg5-inhibitor STLC. Inhibition of Eg5 both blocks centrosome separation in prophase (Bertran et al., 2011; Tanenbaum et al., 2008; Whitehead and Rattner, 1998; Woodcock et al., 2010), and prevents spindle assembly during prometapahase across species (Blangy et al., 1995; Mayer et al., 1999; Sawin et al., 1992; Whitehead and Rattner, 1998). Upon release from the STLC-induced mitotic arrest, the spindle becomes bipolar, which allowed us to easily follow spindle assembly quantitatively, although there is an STLC-induced increase in merotelic kinetochore-microtubule attachments, which has the potential to interfere with the normal dynamics of spindle assembly (Lampson et al., 2004). Maximal spindle elongation took place within 20 minutes after the onset of centrosome separation. To analyze the dynamics of spindle formation, we aligned the cells with respect to the time at which centrosome separation occurred, defined as the last point before which spindle length exceeds 5 µm. Following STLC washout, the spindle length of control cells elongated and reached a steady-state size of 12.1 ± 1.7 µm within 20 minutes of centrosome separation. The spindle also rapidly reached its steady-state width of 9.5 ± 0.6 µm.

**Fig. 1.**
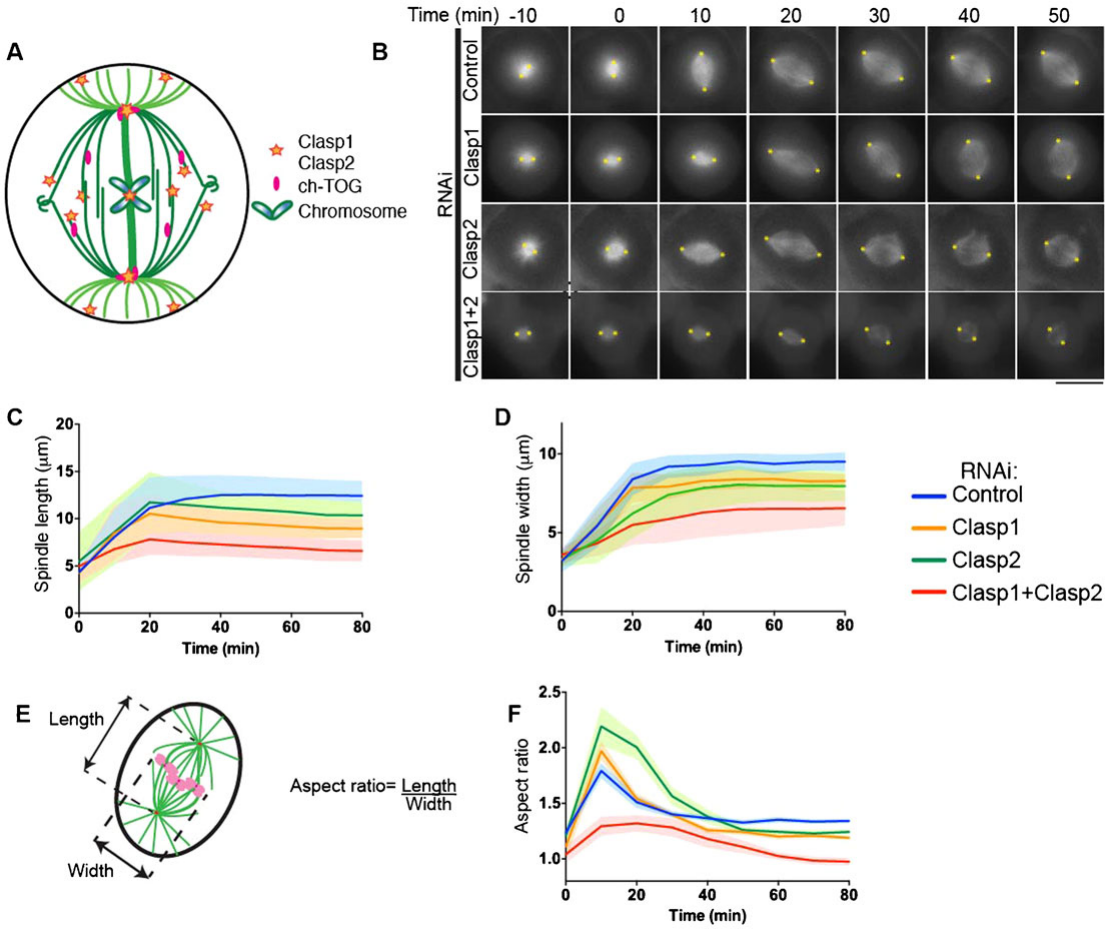
Clasp1 and Clasp2 depletion triggers a biphasic spindle assembly phase and changes in spindle geometry. (A) Schematic diagram showing the localization of ch-TOG, Clasp1 and Clasp2 in a mitotic cell. The cell boundaries are represented in black, K-fibers, spindle microtubules and astral microtubules are represented in dark, medium and light green respectively. (B) Time-lapse imaging of U2OS cells expressing mCherry-tubulin after a STLC washout. Cells were treated with Control, Clasp1, Clasp2 and Clasp1/Clasp2 siRNA for 48 hours before imaging. The yellow asterisks represent the marking of the spindle pole used in the measurements of spindle length, using OMERO. (C,D,F) Graphs representing the average spindle length, width and aspect ratio and the corresponding SD, defined in panel E during elongation for each condition described in panel B. Scale bar: 10 µm.

Ch-TOG, Clasp1, and Clasp2 influence microtubule length by controlling microtubule plus-end dynamics (supplementary material Fig. S1A,B) (Al-Bassam et al., 2012; Brouhard et al., 2008; Komarova et al., 2009; Maffini et al., 2009; Maiato et al., 2003; Mimori-Kiyosue et al., 2005). In addition to their localization to microtubule plus ends, ch-TOG and the Clasp proteins localize to subcellular structures including the kinetochore and the centrosome to direct correct chromosome segregation (Fig. 1A) (Dionne et al., 2000; Maiato et al., 2003; Pereira et al., 2006). To determine whether these microtubule regulators control spindle geometry, we next examined bipolar spindle elongation in absence of ch-TOG, Clasp1, or Clasp2. Upon STLC washout, ch-TOG depleted cells failed to separate their centrosomes, despite the presence of short microtubules (data not shown).

Upon depletion of Clasp1, Clasp2, or Clasp1/Clasp2 co-depletion, the steady-state spindle length was shorter following STLC washout than in control cells (Fig. 1B,C; supplementary material Fig. S1C). Spindles displayed a biphasic spindle assembly in the absence of Clasp1, Clasp2, or Clasp1/Clasp2 (Fig. 1B,C; supplementary material Fig. S2A–D, Fig. S3). During the elongation phase, the spindle length reached a maximum of 10.8 ± 1.8 µm, 12.2 ± 2.6 µm, and 7.1 ± 1.9 µm for Clasp1, Clasp2, and Clasp1/Clasp2 depletion, respectively. This elongation phase was then followed by a contraction phase to a steady-state length that was significantly shorter than control cells (8.7 ± 1.1 µm, 9.3 ± 1.2 µm, and 6.7 ± 0.9 µm for Clasp1, Clasp2, and Clasp1/Clasp2 depletion, respectively, supplementary material Table S1). Spindle width was also reduced in Clasp1, Clasp2, and Clasp1/Clasp2 depleted cells (8.3 ± 0.4 µm, 8.0 ± 0.9 µm, 6.6 ± 1.1 µm, respectively, Fig. 1D) compared to control cells (9.5 ± 0.6 µm). However, in contrast to spindle length, the increase of spindle width in Clasp-depleted cells was monophasic.

We next analyzed the shape of the spindle defined as the aspect ratio or length-to-width ratio, during spindle elongation (Fig. 1E). The aspect ratio of the spindle varied dramatically in absence of Clasp1 and notably Clasp2, revealing a plasticity of spindle shape during spindle elongation (Fig. 1F). At metaphase, Clasp1 and Clasp2 single depletions affected spindle shape, with a rounder shaped spindle compared to control cells (Fig. 1F). During spindle elongation, the aspect ratio of Clasp1/Clasp2 co-depleted spindles remained close to 1. These Clasp1/Clasp2 co-depleted spindles lost their diamond-like shape, with the spindle width nearing spindle length at steady-state equilibrium. Thus, in absence of Clasps, spindle shape was not maintained during the scaling of spindle length. Taken together, Clasps are critical to ensure the stabilization of the steady-state spindle length and the correct diamond-shaped spindle geometry.

### Kid antagonizes Clasps during spindle elongation

**Fig. 2.**
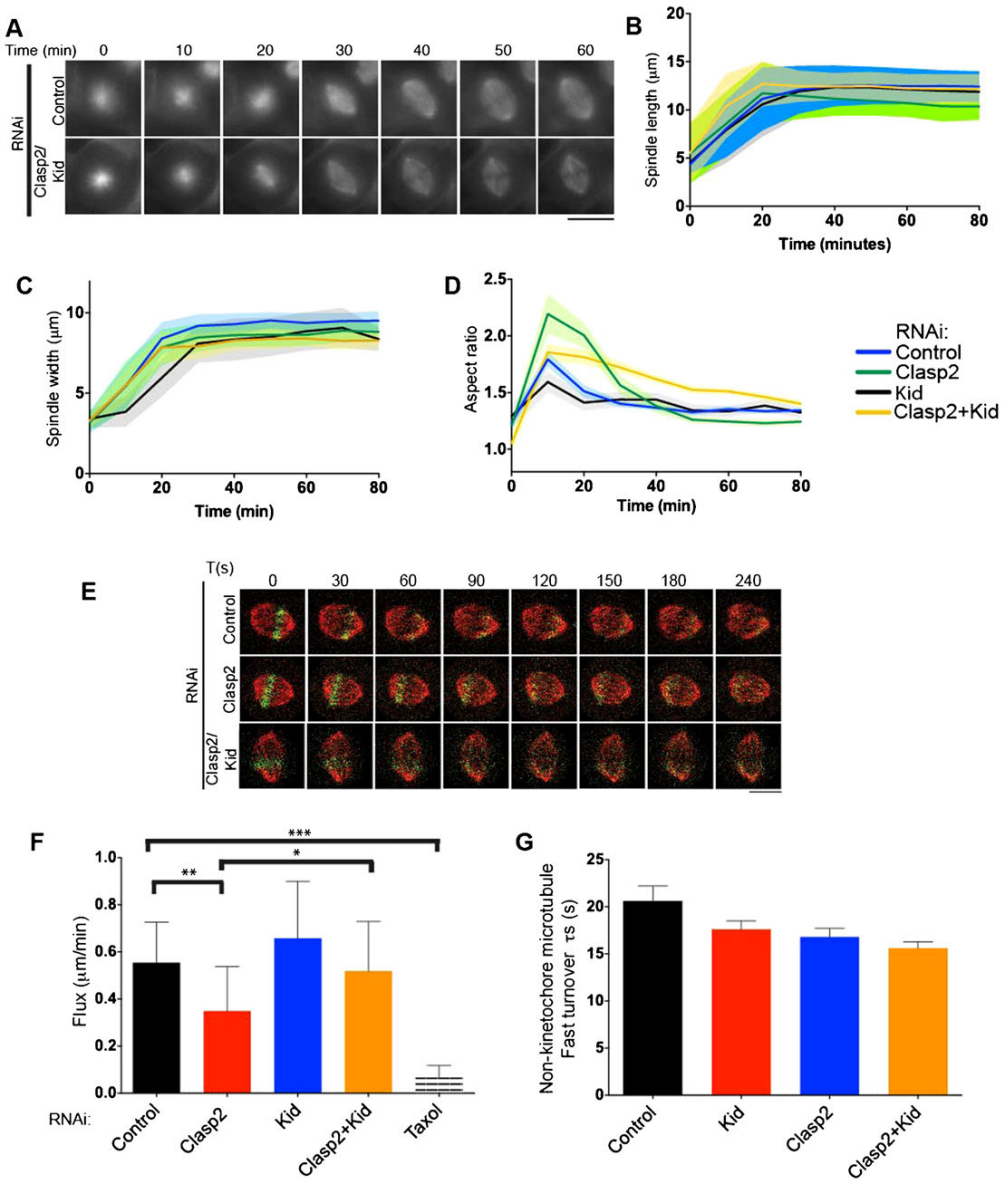
The chromokinesin Kid opposes Clasp1 and Clasp2 during spindle formation. (A) Time-lapse imaging of U2OS cells expressing mCherry-tubulin after a STLC washout. (B–D) Graphs representing the average spindle length, width and aspect ratio and the corresponding SD, during elongation for each condition described in panel B. (E) Time-lapse imaging of U2OS cells expressing mCherry-tubulin and PA-GFP-tubulin before and at 30 s intervals after photoactivation at 405 nm. (F,G) Quantification of microtubule poleward rate + SD and fast turnover + sem in cells treated with Control, Kid, Clasp2 or Clasp2/Kid siRNA. *, ** and *** represent a P<0.5, P<0.01 and P<0.001 respectively. Scale bars: 10 µm.

Polar ejection forces contribute to chromosome alignment by pushing chromatin away from the spindle poles during prometaphase (Magidson et al., 2011). The kinesin-10 family chromokinesin Kid is the major contributor to polar ejection forces (Bieling et al., 2010; Cane et al., 2013; Stumpff et al., 2012). We hypothesized that polar ejection forces could play a role in spindle shape. Kid depletion alone did not alter spindle length or width significantly by live-cell imaging (12.8 ± 1.7 µm and 8.8 ± 1.2 µm, respectively), in agreement with previous work (Stumpff et al., 2012), although a study measuring spindle length in Kid-depleted cells in fixed cells reported a small decrease in length (Fig. 2A–C) (Tokai-Nishizumi et al., 2005). The final spindle width was however reached later than that of control cells, due to reduced polar ejection forces that position chromosomes during prometaphase (supplementary material Fig. S2E,F) (Magidson et al., 2011). As Kid interacts with CLASP (Patel et al., 2012), we also tested whether Kid acts synergistically with Clasps during bipolar spindle formation. When Kid and Clasp2 were co-depleted, spindle length was restored to 12.7 ± 1.2 µm and spindle width was not significantly modified, averaging 8.5+0.9 µm (Fig. 2A–C; supplementary material Table S1). Under these conditions, Kid and Clasp2 were efficiently co-depleted (supplementary material Fig. S1E). We also observed a similar rescue in spindle length following the co-depletion of Kid and Clasp1 (11.5 ± 1.5 µm; supplementary material Fig. S1F; Table S1). Interestingly, the spindles did not contract after maximal elongation in Clasp1 or Clasp2 and Kid co-depleted cells, leading to the higher aspect ratio of Clasp2/Kid spindles (Fig. 2D). Our data suggest that Kid-mediated polar ejection forces on their own do not alter the diamond-shape spindle geometry, but that Clasp1 and Clasp2 have a functional and antagonistic relationship with Kid for regulating spindle geometry.

### *Clasp*s and Kid play antagonistic roles on kinetochore microtubule dynamics to regulate spindle length

At kinetochores, Clasps generate poleward flux on kinetochore-fibers and increase plus end tubulin turnover (Maffini et al., 2009; Maiato et al., 2003). Kid depletion restored a monophasic elongation of the spindle in each individual Clasp depleted condition (Fig. 2A,B; supplementary material Fig. S1F). Thus, we next focused on analyzing the functional relationship between Kid and Clasp2. To test whether Kid could alter spindle flux in absence of Clasp2, we measured microtubule poleward flux in a U2OS cell line expressing mCherry-tubulin and photoactivatable-GFP (PA-GFP)-tubulin. We first determined the flux rate in cells individually depleted for Clasp2 or Kid. Microtubule flux in Clasp2-depleted cells was significantly reduced (0.4 ± 0.04 µm/min, mean ± sem) relative to control cells (0.6 ± 0.03 µm/min), whereas Kid-depleted cells did not display significant changes in the flux rate (0.7 ± 0.04 µm/min; Fig. 2E,F), consistent with previous results (Maffini et al., 2009; Wandke et al., 2012). In contrast, we found that the flux rate was significantly higher in Clasp2/Kid co-depleted cells (0.5 ± 0.04 µm/min) compared to Clasp2 depleted cells and was comparable to control cells (Fig. 2E,F). To test whether Kid and Clasp2 are acting on kinetochore-fibers or interpolar spindle microtubules, we measured microtubule plus end turnover, which appears as the decay of fluorescence intensity of the activated PA-GFP-tubulin mark. We found that fast spindle microtubule turnover at plus ends was not affected by Clasp2 or Kid depletion, suggesting the slower population of kinetochore-fiber microtubules might be modified (Fig. 2G). We could not measure with confidence the K-fiber turnover in our cell line, which did not allow us to test this hypothesis. Also, microtubule flux can be regulated both by minus and plus ends of microtubules in the spindle. Since spindle flux rate was affected by Clasp2 but spindle microtubule plus end turnover rate was not, our result indicates that Clasp2 may play its major role at the minus end of spindle microtubules as well as at kinetochore-fibers plus ends. Future work should test this model. In total, Kid antagonizes Clasp2 to restore spindle poleward flux and consequently restore metaphase spindle length.

### *Clasp*s act on kinetochore-fibers and spindle microtubules to stabilize the spindle shape and length

**Fig. 3.**
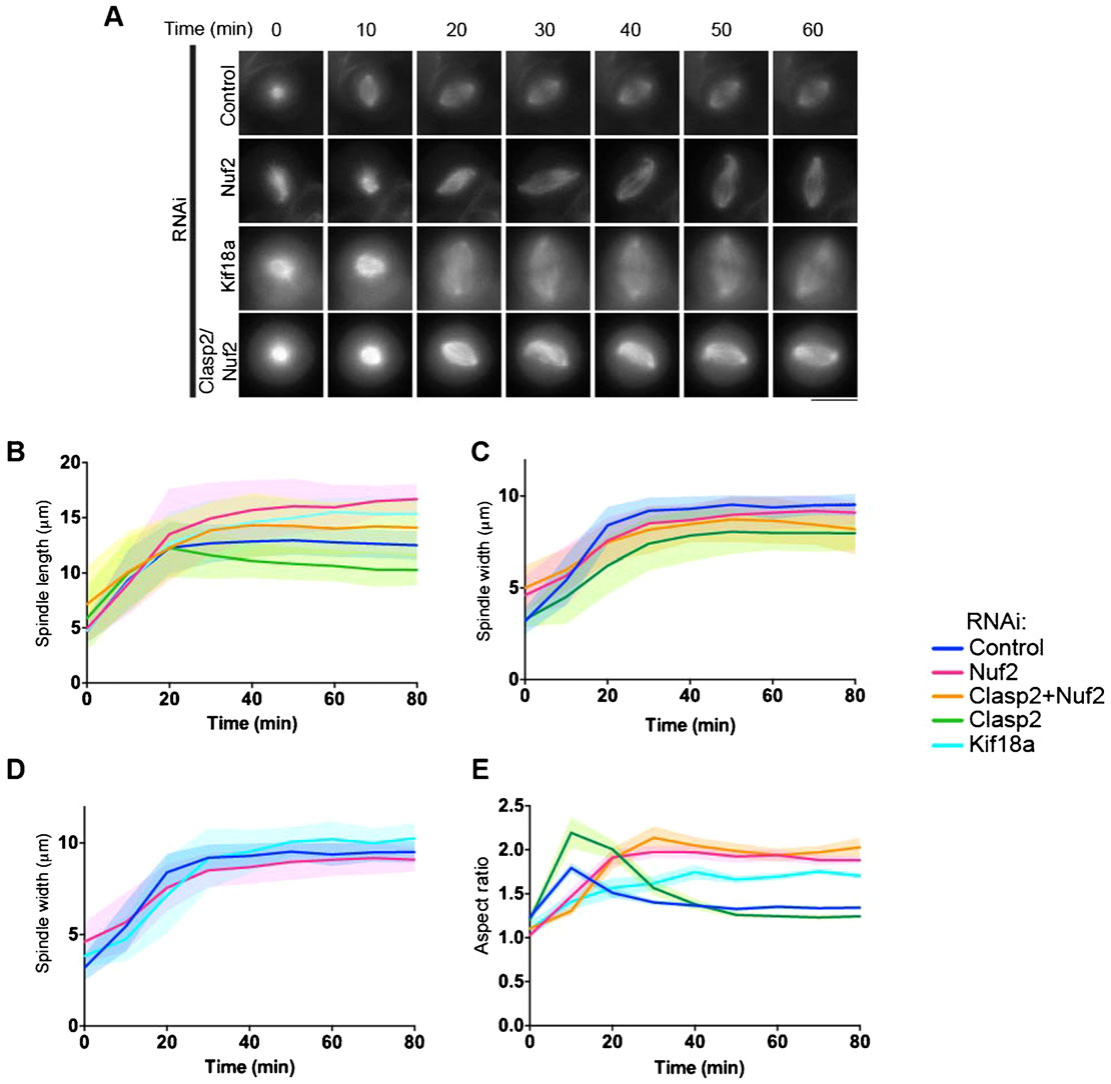
Clasp1 and Clasp2 act on kinetochore-fibers to stabilize the steady-state metaphase spindle. (A) Time-lapse imaging of U2OS cells expressing mCherry-tubulin after a STLC washout, treated with indicated siRNA. (B–E) Graphs representing the average spindle length, width and aspect ratio and the corresponding SD during elongation for each condition described in panel A. Scale bar: 10 µm.

Clasp1, Clasp2 and Kid may act antagonistically on the microtubule dynamics of kinetochore-fibers. To test whether the spindle contraction and changes in spindle geometry was due to the absence of Clasps at kinetochore-fiber ends, we examined spindle elongation in absence of kinetochore-fibers by depleting the kinetochore protein Nuf2. We first depleted Nuf2 alone, which results in abnormally long spindles (Fig. 3A,B; supplementary material Fig. S2G,H) consistent with prior work (DeLuca et al., 2006). Upon Clasp2/Nuf2 co-depletion, the biphasic spindle assembly behavior was suppressed (Fig. 3A,B; supplementary material Fig. S2I,J). The spindle reached a steady-state spindle length of 12.8 ± 2.3 µm, similar to control spindles (supplementary material Fig. S1C). We propose that upon end-on attachment of kinetochore-fibers to kinetochores, kinetochore-fibers generate an inward pulling force to stabilize the metaphase spindle. In the absence of Clasps, the shorter centrosome and spindle-generated interpolar microtubules determine the steady-state spindle length. Taken together, our data suggest that Clasp1 and Clasp2 act both on kinetochore-fibers, to enable a monophasic spindle elongation, and on spindle microtubules to set the steady-state spindle length.

### Kinetochore-fibers promote a diamond-shaped spindle

In the absence of kinetochore-fibers, there are no inward pulling forces acting at kinetochores to maintain the steady-state metaphase spindle architecture. We found that spindle width decreased in absence of kinetochore-fibers (8.7 ± 1.2 µm and 9.0 ± 1.0 µm for Nuf2/Clasp2 and Nuf2-depleted cells; Fig. 3A,C). To test how kinetochore-fiber dynamics modulate spindle shape, we generated long spindles without disrupting kinetochore-fibers by depleting the microtubule depolymerase Kif18a. Kif18a-depleted spindles were abnormally long, averaging 15.9 ± 1.3 µm (Fig. 3B; supplementary material Fig. S2G) (Mayr et al., 2007; Savoian and Glover, 2010; Syrovatkina et al., 2013). Unlike Nuf2 depletions, spindle width also increased to 10.2 ± 0.8 µm following Kif18a depletion (Fig. 3D; supplementary material Fig. S2H). Consequently, the aspect ratio was high in all long spindles, but was the most elevated in spindles lacking kinetochore-fibers, because width did not increase with increasing length. Thus, the dynamics of kinetochore-fibers play a role in shaping the spindle and maintaining a correct length-to-width ratio, which is essential to capture and align chromosomes correctly during metaphase.

### Spindle shape is determined by spindle length

**Fig. 4.**
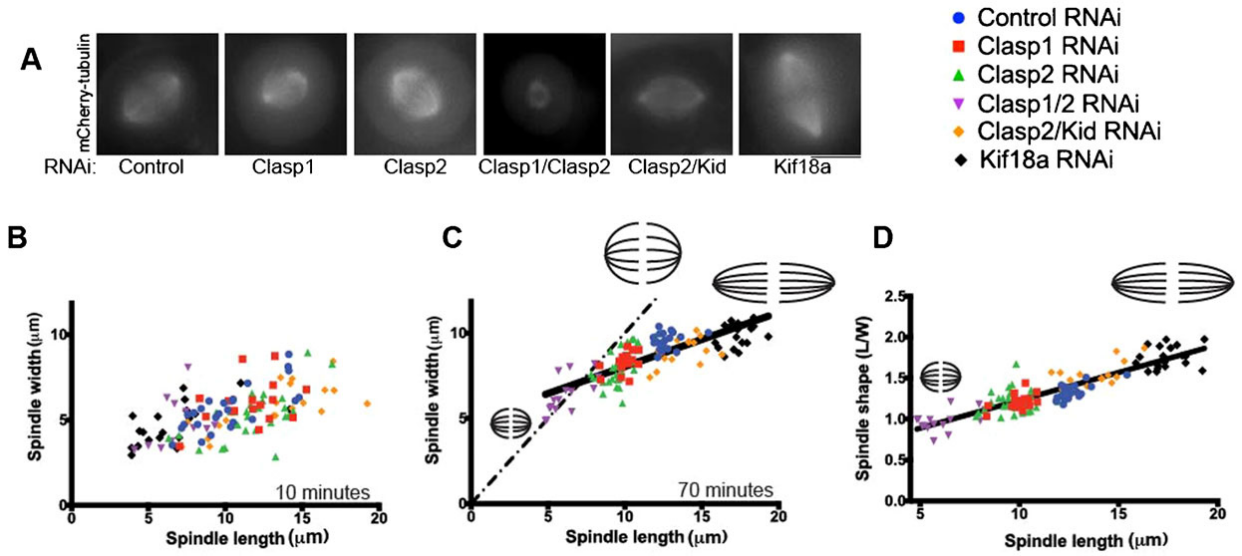
Spindle shape is modulated by regulators of microtubule dynamics. (A) Representative images of spindles for cells depleted with indicated siRNA. Graphs representing spindle width versus spindle length for conditions in (B) 10 minutes and (C) 70 minutes after centrosome separation for conditions described above. The dashed line represents theoretical isotropic scaling. (D) Graph representing spindle aspect ratio versus spindle length for conditions in panel A. Raw data (118 points) were fitted to a linear regression function (R^2^ = 0.80). Scale bar: 10 µm.

Above, we found that changes in spindle length and width alter spindle geometry (Fig. 4A). Therefore, we sought to determine whether there are any general rules that govern spindle shape. To test this, we analyzed the aspect ratio of measured spindles independently of the specific perturbation when all microtubule populations were present. Spindle width rapidly reached a steady-state equilibrium around 40 minutes after the initiation of elongation, regardless of the specific perturbation analyzed (Fig. 1E, Fig. 2D, Fig. 3E). During the early stages of spindle elongation, spindle length and width were not correlated (Fig. 4B). However, at steady-state, there was a linear correlation between the aspect ratio and spindle length (Fig. 4C,D). Longer spindles had a high aspect ratio, whereas the shortest spindles appeared circular, with an aspect ratio nearing 1. This is due to the fact that width and length do not scale with the same factor. Overall, our data suggest that spindle width and length are coupled anisotropically. Previous work has shown that spindle length and cell size are coupled (Good et al., 2013; Hazel et al., 2013). Our data demonstrate that spindle geometry is constrained, with spindle shape being linearly correlated with the length of the spindle.

### Biological significance of spindle scaling

Our geometric and kinetic measurements show that there is a linear relationship between spindle length and width, irrespective of the spindle components. Such a coupling has also been observed with increasing concentrations of XMAP215 and in *C. elegans*, where width and length scaled with each other (Hara and Kimura, 2013; Reber et al., 2013). Taken together, this suggests a universal behavior for the relationship between spindle width and spindle length. We propose that this geometrical coupling of spindle shape and length plays an important role in promoting correct chromosome segregation. During mitosis, the spindle needs to satisfy a minimum width so that it can capture and biorient chromosomes at the metaphase plate. Indeed, the inability to correctly scale the spindle geometry in tetraploid cells, where spindle width increases, but spindle length remains the same, leads to genomic instability (Storchová et al., 2006). Incorrect kinetics of spindle geometry, through multipolar configurations, also cause merotelic attachments and lagging chromosomes in human cells (Ganem et al., 2009). Shorter spindles must remain wide to maximize the spatial ability to achieve chromosome capture and alignment. However, the geometry of the spindle has to be optimized to maximize the utilization of available and rate-limiting cellular resources, such as tubulin and microtubule-associated proteins. Thus, an increased spindle length results in moderate spindle widening and an oblong-shaped spindle, as this is sufficient to promote efficient chromosome congression. Spindle shape is therefore determined by its functional requirements and by the availability of its individual building blocks for self-organization.

Spindle composition changes during development and between cell type to regulate spindle length (Loughlin et al., 2011; Wilbur and Heald, 2013). During development, cytoplasmic volume and cell boundaries play an important role in regulating spindle shape and length (Good et al., 2013; Hazel et al., 2013). However, there are intrinsic factors that, in an unbound regime, set the upper limit of spindle size and the spindle geometry (Good et al., 2013; Wühr et al., 2008). We demonstrated that spindle composition also affects spindle shape in a length-dependent manner. Thus, the cytoplasmic availability and regulation of the key regulatory microtubule players dictates the overall spindle length and shape. By ensuring a key spindle-length regulator is present in rate-limiting amounts or absent, the cell can modify the shape of the spindle. In total, these changes in spindle composition give rise to anisotropic scaling of the spindle and reveal a mechanism that generates spindle shape diversity.

During the early stages of development in *C. elegans*, anisotropic spindle scaling is also observed with decreasing cell volume (Hara and Kimura, 2013). The geometry of the spindle may also vary with the number of chromosomes that need to be segregated and the type of kinetochores they contain (Dinarina et al., 2009). Extrinsic factors, such as mechanical stress on the spindle, also change the spindle shape (Dumont and Mitchison, 2009a). Future work should address how spindle shape affects spindle mechanics and functions to ensure accurate chromosome segregation.

## MATERIALS AND METHODS

### Molecular biology and cell culture

The U2OS mCherry-tubulin PA-GFP-tubulin cell line was maintained in DMEM (Lonza) supplemented with 10% FBS, penicillin/streptomycin (Gibco) at 37°C in a humidified atmosphere with 5% CO_2_. Cells were plated on 18-mm glass coverslips coated with poly-L-lysine (Sigma–Aldrich) for immunostaining or 35-mm glass bottom microwell dishes (MatTek) for time-lapse imaging. RNAi experiments were conducted using RNAi MAX transfection reagent (Invitrogen) according to the manufacturer's guidelines. Previously published siRNA oligos were used to deplete Kif18a (Stumpff et al., 2008), Clasp1 and Clasp2 (Mimori-Kiyosue et al., 2005), Kid (Tokai-Nishizumi et al., 2005) and Nuf2 (DeLuca et al., 2006). For experiments with small molecule inhibitors, the proteasome inhibitor MG132, the Eg5 inhibitor STLC and taxol were used at a final concentration of 10 µM, 10 µM and 1 µM respectively.

### Immunofluorescence and live-cell imaging microscopy

Immunofluorescence and live-cell imaging was performed as described (Welburn et al., 2010). Images were acquired on a DeltaVision Core microscope (Applied Precision). On average, experiments were repeated 3 times (supplementary material Fig. S1D). For spindle length measurement, we measured the distance between the two microtubule organizing centers marked by maximum fluorescence of tubulin. The width was measured by taking the cross-section at the center of the spindle, marked by a decrease in tubulin density, due to chromosome mass. Spindle elongation alignment was performed using a MATLAB-based script. Data are reported as mean ± SD, unless mentioned. Statistical analysis using an ANOVA with Tukey's test was performed using GraphPad Prism.

### Photoactivation experiments

Images were acquired on a Leica SP5 Spinning Disc Confocal microscope using a 63×/1.4 NA objective lens, with an additional 5× zoom. A 1.2 µm × 12.6 µm region of photoactivation was manually specified for each spindle prior to imaging. 5 pre-activation frames were acquired followed by a single 0.648 s pulse from a 405 nm laser to activate the GFP-α-tubulin. Post-activation images were acquired every 10 seconds for 300 seconds. Collected images were imported and stored onto an OMERO server (Allan et al., 2012). Cells that underwent anaphase or with low signal-to-noise ratios were discarded from the analysis. For each candidate cell, the centrosome positions at each time point was marked as regions of interests using the built-in Measurement Tool in the OMERO.insight client. Statistical analysis using an ANOVA with Tukey's test was performed using GraphPad Prism.

Photoactivation analysis was performed using MATLAB (Mathworks) and the OMERO.matlab bindings as follows. For each time point, the image pixel data and the centrosome position were read from the server. The intensity profile was computed alongside the centrosome–centrosome line using a Radon transform. This intensity profile was background corrected by thresholding the low-pass filtered image, computing its Radon transform and subtracting it from the uncorrected intensity profile (supplementary material Fig. S4A,B). Photoactivation heat maps are generated by concatenating these background-subtracted intensity profiles (supplementary material Fig. S4C). For each time point, the corrected intensity profile was fitted using a 1D Gaussian mixture model (supplementary material Fig. S4D,E). The amplitude and the position of these models yield the signals and position of the photoactivated spindle as well as the signal intensity background spindle.

MT flux speed was quantified by linearly fitting the position of the photoactivated signal as a function of time on the first 150 seconds (supplementary material Fig. S4F). MT turnover was quantified from the decay of the corrected normalized photoactivated spindle intensity. Background correction was performed by subtracting the background spindle signal intensity from the photoactivated spindle signal at each time point. The background-subtracted intensity was then normalized by the value of the first time point. Photobleaching correction was then performed using the averaged background-subtracted intensities from a series of control cells treated with 1 µM of taxol. Each fully corrected intensity time-series was individually fitted by a double-exponential curve I = P_f_ exp(−t/τ_f_)+P_s_ exp(−t/τ_s_), where I is the proportion of the initial fluorescence intensity, P is the proportion of fluorescence decay due to the fast (f) or slow (s) process, τ is the time constant for fluorescence decay of the fast (f) or slow (s) process, and t is time (supplementary material Fig. S4G; Table S2). The goodness-of-fit was estimated by computing the coefficient of determination from the norm of the residuals. For each condition, the mean flux and slow and fast turnover times were computed by averaging the fitted values of individual series. We did not report the slow turnover because of the heterogeneity of the K-fiber measurements.

Scripts are available at http://welburn.bio.ed.ac.uk/resources.

### Abbreviations

PA: photoactivatable; STLC: S-Trityl-L-cysteine.

## Supplementary Material

Supplementary Material
