# Supplementary material for: Length-dependent anisotropic scaling of spindle shape
Source: Biol Open. 2014 Nov 21;3(12):1217–23. doi: 10.1242/bio.201410363 (PMC4265759; doi:10.1242/bio.201410363)
Supplement: Supplementary Material [file supp_3_12_1217__index.html]

Length-dependent anisotropic scaling of spindle shape — Length-dependent anisotropic scaling of spindle shape — Supplementary Material 

# Length-dependent anisotropic scaling of spindle shape

## bio.201410363 Supplementary Material

**Files in this Data Supplement:**

- Supplementary Material - Sarah Young et al. doi: 10.1242/bio.201410363
