# Supplementary material for: Length-dependent anisotropic scaling of spindle shape
Source: Biol Open. 2014 Nov 21;3(12):1217–23. doi: 10.1242/bio.201410363 (PMC4265759; doi:10.1242/bio.201410363)
Supplement: Supplementary Material [file supp_bio.201410363_bio.201410363-s1.pdf]

Biology Open (2014) 000, 1–7 doi:10.1242/bio.201410363

Supplementary Material
Sarah Young et al. doi: 10.1242/bio.201410363

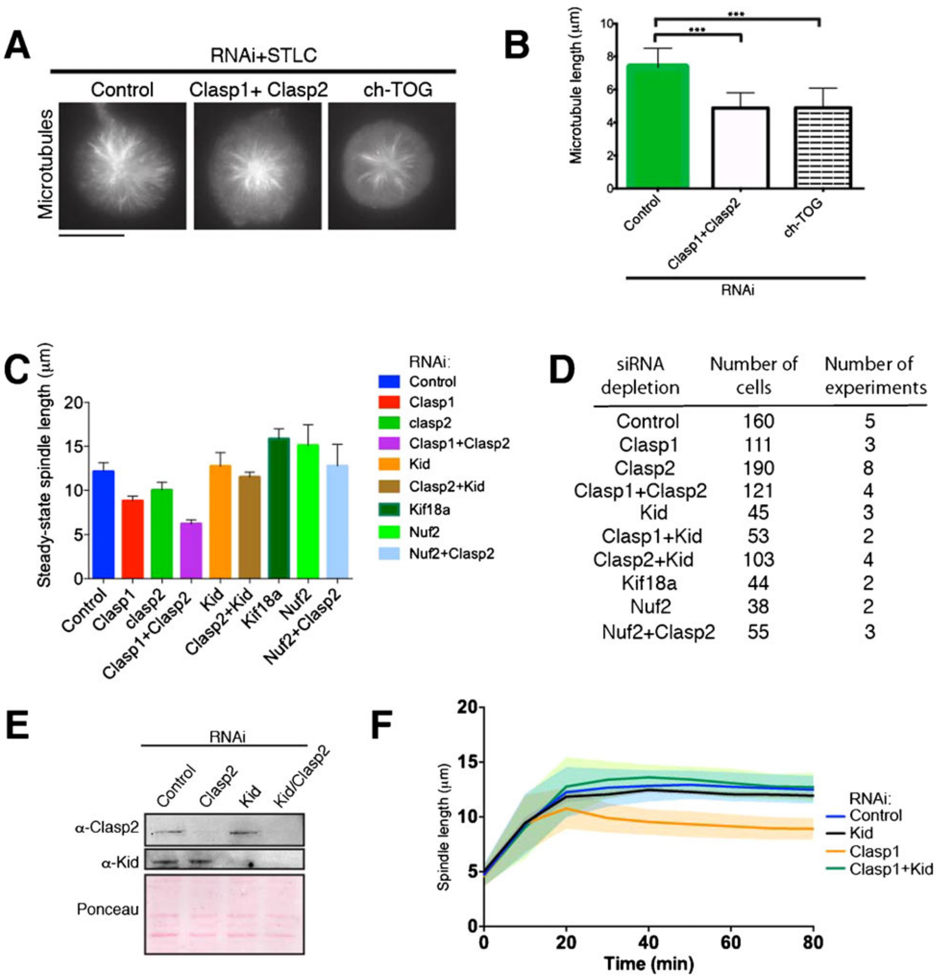

siRNA depletion	Number of cells	Number of experiments
Control	160	5
Clasp1	111	3
Clasp2	190	8
Clasp1+Clasp2	121	4
Kid	45	3
Clasp1+Kid	53	2
Clasp2+Kid	103	4
Kif18a	44	2
Nuf2	38	2
Nuf2+Clasp2	55	3

Fig. S1. Regulators of microtubule ends influence spindle metaphase length differentially. (A) Images of U2OS cells depleted with indicated siRNA and treated with STLC. (B) Bar graph showing the quantified average microtubule length for microtubules radiating from the centrosome for depletions in panel A (mean ± SD). (C) Average spindle length and SD at steady-state of U2OS cells expressing mCherry-tubulin treated with the indicated siRNA. (D) Table representing the number of cells and experiments performed monitoring spindle elongation. (E) Western blot showing levels of Kid and Clasp2 after Kid/Clasp2 co-depletion for 48 hours. (F) Graph representing the average spindle length and SD during elongation for each indicated condition.

Biology Open (2014) 000, 1–7 doi:10.1242/bio.201410363

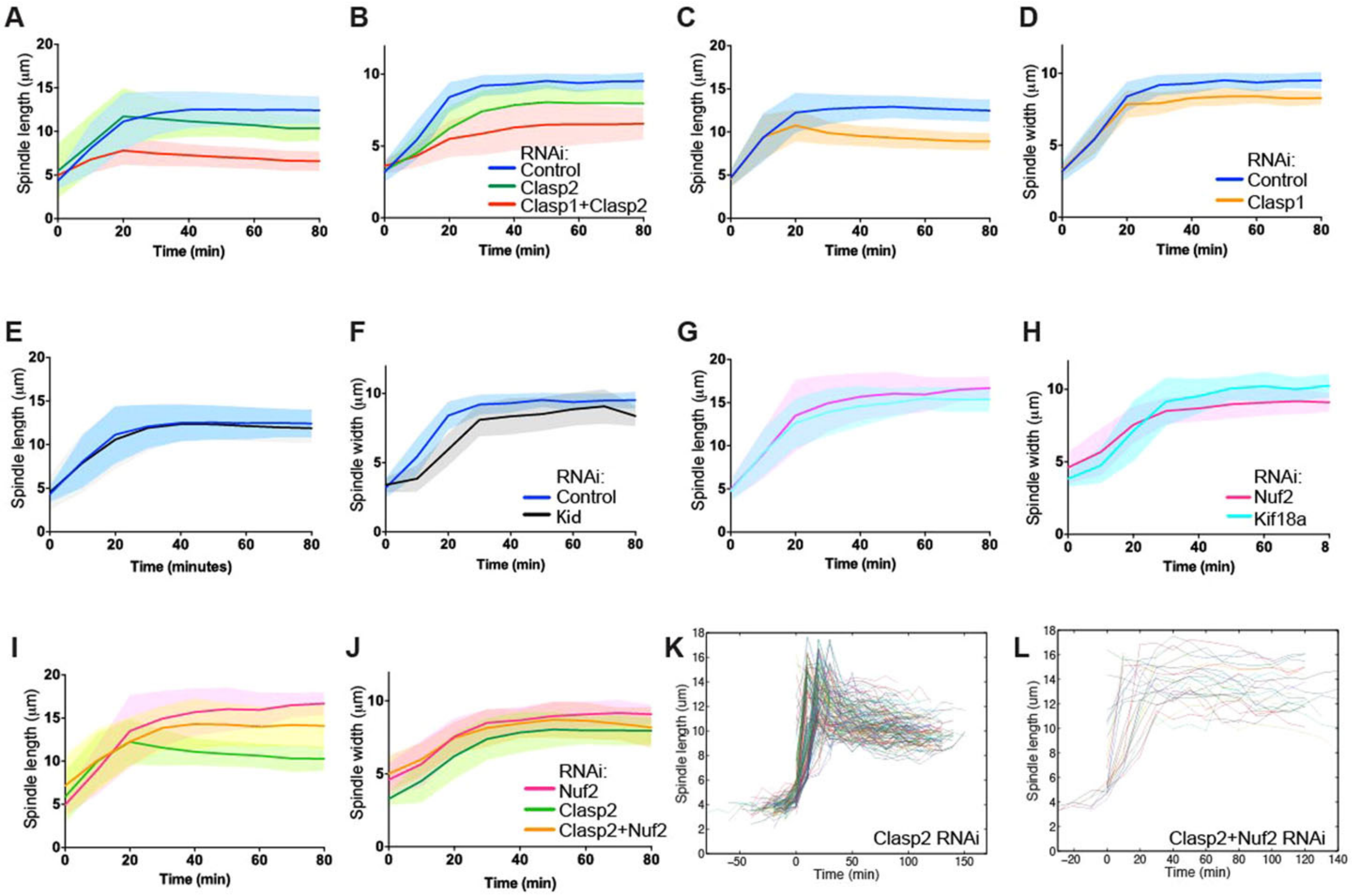

Fig. S2. Pairwise comparison of the dynamics of spindle assembly. Pairwise comparison graphs representing the average spindle length (A,C,E,G,I) and width (B,D,F,H,J) and the corresponding SD, of U2OS cells expressing mCherry-tubulin during spindle elongation after STLC washout. Cells were treated with the indicated siRNA for 48 hours before imaging. (K,L) Representative graphs for the individual spindle elongation profiles for each cell, aligned with respect to the initiation of spindle elongation for Clasp2 and Clasp2/Nuf2 depleted cells.

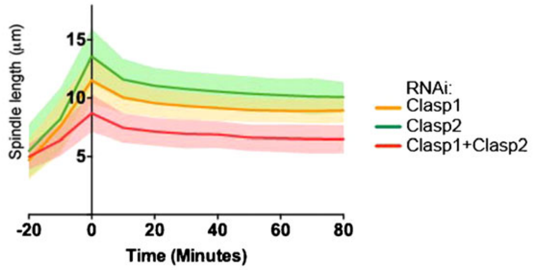

Fig. S3. Dynamics of spindle contraction for CLASP depletions. Graph representing the average spindle length and the corresponding SD as a function of time of Clasp1, Clasp2 and Clasp1+2 depleted U2OS cells expressing mCherry-tubulin during spindle elongation after STLC washout. Individual spindle elongation profiles were aligned with respect to the time at which spindles reached their maximum length.

Biology Open (2014) 000, 1–7 doi:10.1242/bio.201410363

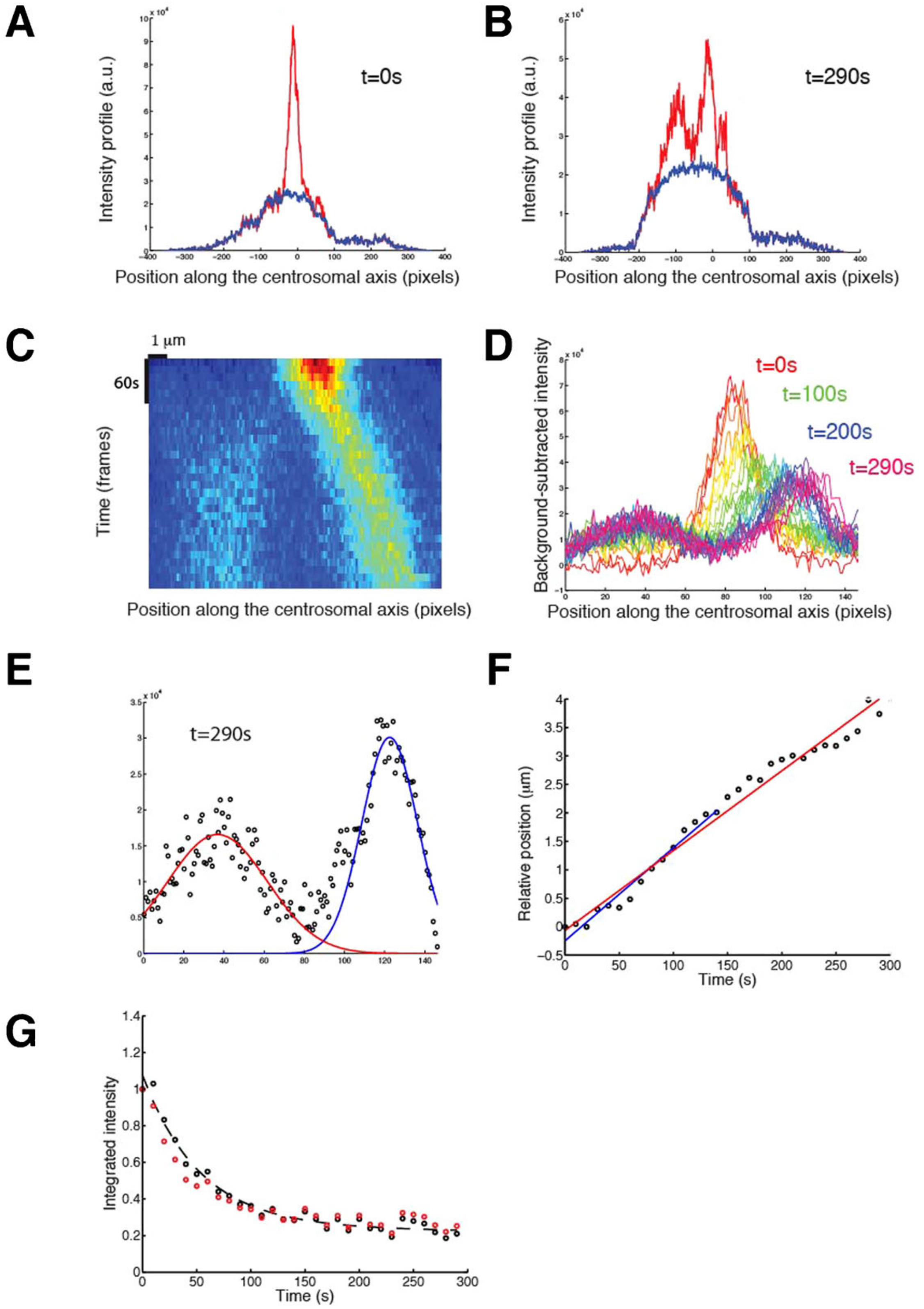

Fig. S4. Photoactivation analysis process of a representative cell depleted with Control siRNA for 48 hours. (A,B) Raw intensity profile computed by a Radon transform alongside the centrosome–centrosome axis at times (A) t=0 s and (B) t=290 s. Blue line corresponds to the Radon transform of the background. (C) Heat map of the background subtracted intensity profile as a function of time. (D) Overlay of the background-subtracted intensity profiles as a function of the centrosome–centrosome distance for all measured times. (E) Fit of the background subtracted intensity profile by a 1D Gaussian mixture model at t=0 s and t=290 s. The blue Gaussian fit corresponds to the photoactivated spindle. (F) Evolution of the relative distance of the center of the photoactivated band to the centrosome position as a function of time. Red and blue lines show the output of a linear regression over the whole time range and half time range respectively. (G) Evolution of the background spindle corrected (black points) and fully corrected (red points) photoactivated intensity as a function of time. Dashed line indicates the output of the non-linear regression using a double exponential model.

Biology Open

Biology Open (2014) 000, 1–7 doi:10.1242/bio.201410363

Table S1. Parameters of the microtubule turnover fit for the photoactivation data

	pf	tf (s)	ps	ts (s)
**Control RNAi**				
Average fit	0.770281	20.6079	0.229719	178.58
Average fit SE	0.0398843	1.67735	0.0191718	113.999
**Clasp2 RNAi**				
Average fit	0.850469	17.583	0.149531	297.475
Average fit SE	0.0235605	0.937045	0.0778402	411.734
**Kid RNAi**				
Average fit	0.960666	16.773	0.0393339	143.484
Average fit SE	0.0299124	0.893069	0.0155161	317.791
**Clasp2+Kid RNAi**				
Average fit	0.944515	15.6438	0.0554848	86.1712
Average fit SE	0.0305378	0.723418	0.0262876	70.6967

Table S2. P-values calculated from an ANOVA Tukey's test for the differences between spindle lengths for different depletion conditions at steady-state metaphase length

RNAi	control	clasp1	clasp1+2	clasp1+kid	clasp2	clasp2+kid	clasp2+nuf2	kid
control								
clasp1	7.00E–07							
clasp1+2	7.00E–07	7.00E–07						
clasp1+kid	1	7.00E–07	7.00E–07					
clasp2	7.00E–07	7.01E–07	7.00E–07	7.00E–07				
clasp2+kid	0.781214	7.00E–07	7.00E–07	0.94895	7.00E–07			
clasp2+nuf2	1.05E–05	7.00E–07	7.00E–07	0.0116724	7.00E–07	7.04E–07		
kid	0.849049	7.00E–07	7.00E–07	0.921082	7.14E–07	1	9.46E–07	
nuf2	7.00E–07	7.00E–07	7.00E–07	7.00E–07	7.00E–07	7.00E–07	1.05E–06	7.00E–07

Biology Open
